# B2 Thickness Parameter Response to Equinoctial Geomagnetic Storms

**DOI:** 10.3390/s21217369

**Published:** 2021-11-05

**Authors:** Yenca Migoya-Orué, Katy Alazo-Cuartas, Anton Kashcheyev, Christine Amory-Mazaudier, Sandro Radicella, Bruno Nava, Rolland Fleury, Rodolfo Ezquer

**Affiliations:** 1 The Abdus Salam International Centre for Theoretical Physics (ICTP), Strada Costiera 11, I-34151 Trieste, Italy; yenca@ictp.it (Y.M.-O.); katyalazo@yahoo.com (K.A.-C.); christine.amory@lpp.polytechnique.fr (C.A.-M.); bnava@ictp.it (B.N.); 2 Physics Department, University of New Brunswick, Fredericton, NB E3B 5A3, Canada; anton.kashcheyev@unb.ca; 3 Sorbonne Université, Ecole Polytechnique, Institut Polytechnique de Paris, Université Paris Saclay, Observatoire de Paris, CNRS, Laboratoire de Physique des Plasmas (LPP), 75005 Paris, France; 4 LAB-STICC, UMR6285, Institut Mines-Telecom, CEDEX 3, 29288 Brest, France; rolland.fleury@telecom-bretagne.eu; 5 Laboratorio de Ionósfera, FACET, Universidad Nacional de Tucumán, S. M. de Tucumán 4000, Argentina; rezquer@gmail.com

**Keywords:** ionospheric empirical models, thickness parameter, NeQuick model, total electron content, geomagnetic storms

## Abstract

The thickness parameters that most empirical models use are generally defined by empirical relations related to ionogram characteristics. This is the case with the NeQuick model that uses an inflection point below the F2 layer peak to define a thickness parameter of the F2 bottomside of the electron density profile, which is named B2. This study is focused on the effects of geomagnetic storms on the thickness parameter B2. We selected three equinoctial storms, namely 17 March 2013, 2 October 2013 and 17 March 2015. To investigate the behavior of the B2 parameter before, during and after those events, we have analyzed variations of GNSS derived vertical TEC (VTEC) and maximum electron density (NmF2) obtained from manually scaled ionograms over 20 stations at middle and low latitudes of Asian, Euro-African and American longitude sectors. The results show two main kinds of responses after the onset of the geomagnetic events: a peak of B2 parameter prior to the increase in VTEC and NmF2 (in ~60% of the cases) and a fluctuation in B2 associated with a decrease in VTEC and NmF2 (~25% of the cases). The behavior observed has been related to the dominant factor acting after the CME shocks associated with positive and negative storm effects. Investigation into the time delay of the different measurements according to location showed that B2 reacts before NmF2 and VTEC after the onset of the storms in all the cases. The sensitivity shown by B2 during the studied storms might indicate that experimentally derived thickness parameter B2 could be incorporated into the empirical models such as NeQuick in order to adapt them to storm situations that represent extreme cases of ionospheric weather-like conditions.

## 1. Introduction

It was Alexander von Humboldt, in 1808, who introduced the name of magnetic storm [1] for a phenomenon involving large scale magnetic disturbances associated with northern lights (aurora). For more than two centuries, magnetic storms have been studied and are still a subject of active investigation. In their paper, “what is a geomagnetic storm?”, Gonzalez et al. [2] defined three classes of geomagnetic storms (intense, moderate and small) by using two physical parameters: the interplanetary magnetic field (IMF) Bz component and the disturbed storm time index (Dst). In [3], the authors recalled the classification of magnetic storms since von Humboldt in 1808, with two new classes: great storms and super storms. By comparing two great geomagnetic storms differing only by their season and the onset time of the storm, they showed how these two main parameters help to interpret the observed effects in the ionosphere at middle and low latitudes. At a given location, many parameters influence the variations in the ionosphere observed during a magnetic storm: solar flux, season, storm start time, initial state of the magnetosphere–ionosphere–thermosphere system, atmospheric forcing from low altitudes, etc. During magnetic storms various physical processes can produce positive or negative ionospheric effects, i.e., increase or decrease in the electron density. These are: (1) the thermal expansion of the atmosphere due to Joule heating in the auroral zone, which produces changes in temperature, pressure, wind and composition of the thermosphere; (2) the prompt penetration of magnetospheric convection electric field (PPEF), motional electric fields that penetrate in low latitude ionosphere and magnetosphere after high-latitude convection and (3) the disturbed dynamo electric field (DDEF) produced by storm winds [4].

Theoretical studies made it possible to understand the effect of two parameters that strongly influence the response of the ionosphere. They are time of onset of the geomagnetic storm [5] and season [6]. Fuller-Rowell et al. [5] analyzed the effect of the time at the beginning of a storm. They found that the global wind surge has a preference for the night sector and for the longitude of the magnetic pole and therefore depends on the UT start time of the storm. Fuller-Rowell et al. [6] found that there is a preference for ionospheric negative storm in summer and ionospheric positive storm in winter; this was explained by the prevailing summer–winter thermospheric wind circulation. Prölss [7] found that negative storms are related to the O/N2 ratio. The geomagnetic coordinates are an important factor, and as a consequence the stations located in the North American and Australian sectors are more liable to observe negative storm effects. Stations located in the early morning sector during enhanced substorm activity have a greater chance of observing negative storm effects than those situated in the daytime sector. Prölss [7] also found that there is no correlation between the positive ionospheric storm and changes in the neutral composition. Positive ionospheric storms might be caused by transport of ionization, and furthermore this transport is affected by winds and/or electric fields (PPEF and DDEF).

The effects of the storms in the ionosphere are generally analyzed in terms of the variations of important ionospheric parameters, such as critical frequencies, maximum densities or total electron content (TEC) (see [8] and references therein). Deviations of NmF2 and VTEC under quiet and disturbed conditions over Ilorin have been investigated by [9]. These authors found almost simultaneous but not proportionate variations of these parameters under four different storms, NmF2 being more sensitive to variations than TEC. Most ionospheric empirical models and profilers make use of thickness parameters to describe the shape of the electron density profile. These thickness parameters are defined by empirical relations linked to ionogram characteristics. In the International Reference Ionosphere model (IRI), the F2 bottomside profile is described with a thickness parameter, B0, and a shape parameter, B1 [10]. In [11] there was related an empirical formulation to the inflection point, referred to as the “base point”, that is found in both the experimental and the theoretical profile of the electron density (represented for example by a Chapman or an Epstein layer). The “base point” corresponds to the height of the maximum gradient in the vertical electron density profile below the peak of the F2 layer. This point is considered an “anchor point” in the NeQuick model [12,13] and is used to define a thickness parameter of the bottomside of the electron density profile, which is named B2 and is given in km.

It has to be noted that in the NeQuick formulation the shape of the electron density topside profile depends critically on the value of the bottomside thickness parameter B2. For this reason, it is important to investigate both B2 and VTEC behaviors. In [14] it has been demonstrated that the introduction of ionosonde derived B2 parameter improves the performance of the model in the computation of bottomside TEC reducing the mismodeling. More recently, ref. [15] discussed the performance of different options for thickness parameters in IRI and NeQuick models by comparing modeled and experimental profiles. At the same time, they proposed the use of experimentally derived thickness parameters in the models to better represent and also forecast the electron density profile. The climate behavior of experimentally derived and modelled thickness parameters have been studied regionally and globally [15,16,17,18], while their behavior under storm conditions has not been fully explored.

In this study we selected three storms that occurred in years 2013 and 2015 after magnetic quiet periods, with an onset time between 0 and 6UT and during the same season (equinox) to avoid seasonal variations that play an important role in north–south asymmetries. The selected storms were induced by coronal mass ejections (CMEs), that are defined as large expulsions of plasma and magnetic field from the Sun’s corona. CMEs expand in size as they propagate away from the Sun, and larger ones can reach a size comprising nearly a quarter of the space between Earth and the Sun by the time they reach our planet [19]. For these three storms we analyzed the B2 parameter behavior obtained from profiles derived from ionogram data using the B2 NeQuick analytical formulation; the maximum density of the F2 layer, NmF2, and VTEC derived from GNSS measurements for different longitude sectors. The paper is organized as follows: The second section describes the datasets and data processing techniques, and the third section presents an overview of the geomagnetic storms analyzed. In the fourth section the results are presented, in the fifth the discussion and the sixth is devoted to conclusions.

## 2. Materials and Methods

The main parameters used to analyze the characteristics of the selected geomagnetic storms are given in Table 1. The Sun observations of Sunspot number and F10.7 cm radio flux give the solar regular activity during the periods of the selected storms. The SOHO satellite data allow the determination of the solar event at the origin of the geomagnetic storm. As mentioned above, in the present study, CMEs are at the origin of the storms analyzed. Magnetic indices are used to define the characteristics of the storm in the magnetosphere (Dst for the ring current), in the high-latitude ionosphere (AE and the Polar cap magnetic indices, North and South, give information on the energy transmitted to the ionosphere). The polar cap indices are proxy of the merging field (Em), [20]. The Ap and Am indices can be used to determine the global magnetic activity before and during the magnetic storms.

The main parameters used to analyze the characteristics of the selected geomagnetic storms are given in Table 1. The Sun observations of Sunspot number and F10.7 cm radio flux give the solar regular activity during the periods of the selected storms. The SOHO satellite data allow the determination of the solar event at the origin of the geomagnetic storm. As mentioned above, in the present study, CMEs are at the origin of the storms analyzed. Magnetic indices are used to define the characteristics of the storm in the magnetosphere (Dst for the ring current), in the high-latitude ionosphere (AE and the Polar cap magnetic indices, North and South, give information on the energy transmitted to the ionosphere). The polar cap indices are proxy of the merging field (Em), [20]. The Ap and Am indices can be used to determine the global magnetic activity before and during the magnetic storms.

### 2.1. VTEC Data Maps and from Individual Stations

The relevant VTEC data used are global ionospheric maps (GIMs) produced by the Center for Orbit Determination in Europe (CODE). These VTEC maps are generated at CODE with a 1 h time interval using data from more than 200 GNSS sites of the International GNSS Service (IGS) and other networks. In order to analyze the daily variability of VTEC in different sectors, namely Asian, African and American, VTEC data with 1 h time resolution were extracted from GIMs at the corresponding longitudes.

To obtain VTEC at individual stations, RINEX files from the IGS, UNAVCO, CORS, SONEL and EUREF GNSS networks were processed using the arc-by-arc procedure suggested in [21].

**Figure 1:**
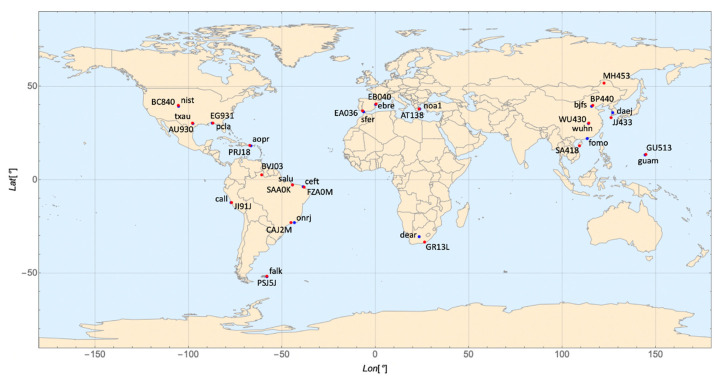
Location of ionosondes (red circles) and GNSS stations (blue circles).

Figure 1 shows the locations of the ionosondes and GPS stations used in this study.

### 2.2. Ionogram Data

The experimental values of the maximum of the electron density derivative with respect to height, (dN/dh)max, were obtained from digisondes’ ionograms from several locations (Figure 1). The ionograms were manually edited using the interactive ionogram analysis tool SAO Explorer [22]. The SAO files were processed with the THTABLE software, a data utility which tabulates and outputs electron density vs. height [23]. The (dN/dh)max value was extracted starting from NmF2 down the profile. Then, B2 values were calculated by applying Equation (1) which is described in detail in [11]:

B2 = (0.385 NmF2)/(dN/dh)max(1)

**Figure 2:**
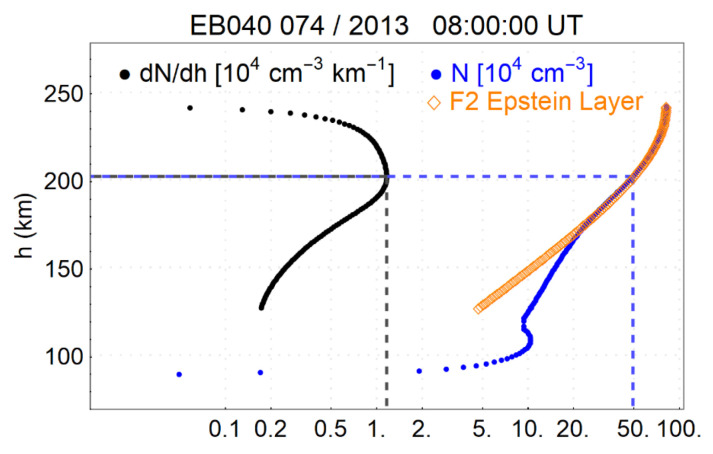
Electron density profile obtained from an ionogram at Ebre (right in blue) and the corresponding dN/dh profile (left). The fit of an Epstein layer on the experimental profile is shown (in orange). Dashed lines indicate the “base point” position.

Figure 2 presents a bottomside profile of the height derivative dN/dh (left) obtained from a diurnal electron density profile at Ebre station (in blue at the right). Over the electron density profile the fit of a theoretical Epstein layer on the F2 layer of the experimental profile is shown (in orange). Both ionogram and theoretical profiles show clearly the presence of a maximum in dN/dh, defined as “base point”, that can be used to define the thickness parameter B2, as explained above.

## 3. Overview of the Three Selected Storms

The three selected storms are those of 17 March 2013, 2 October 2013 and 17 March 2015. The storm of 17 March 2015 was the subject of previous research [3,24]; thus, the context of this storm is not repeated here, and its characteristics are compared to those of the two other storms in Table 2 and Table 3.

**Figure 3:**
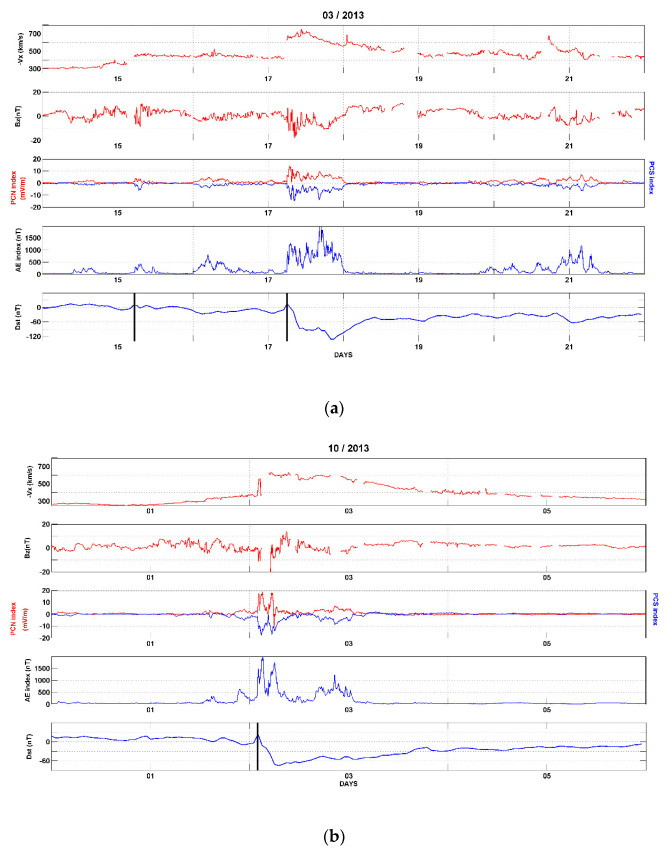
Parameters used to describe the storms (solar wind speed Vx, IMF Bz, polar cap indices, AE and Dst), (**a**) storm of 17 March 2013, (**b**) storm of 2 October 2013. Black vertical lines indicate SSC occurrence.

The monthly averages of the sunspots number and solar radio flux at 10.7 cm wavelength for the months of the magnetic storms (March 2013, October 2013 and March 2015) were 84.4, 107, 82.2 and 117.2, 134.9, 131.6 s.f.u., respectively. The values are comparable, even though the year 2013 is at the peak of the sunspot cycle 24, and the year 2015 is at the beginning of descending phase. The daily values of the magnetic indices Ap for the three days preceding the storms were 4, 6, 10 for the 17 March 2013 storm; 1, 2, 4 for the 2 October 2013 storm and 5, 6, 12 for the 17 March 2015 storm. Thus, the periods preceding the storms can be considered geomagnetically quiet since their Ap values are less than 20 nT. Figure 3a,b show the variability of solar wind conditions and magnetic parameters for the geomagnetic storms of 17 March 2013 and 2 October 2013, from the top to the bottom: Vx, x-component of the solar wind speed, Bz component of IMF, polar cap, AE and Dst magnetic indices. The black vertical bars indicate the sudden storm commencement (SSC). In these figures the same behavior for all the parameters is seen. On 17 March and 2 October 2013 when the CME hits the magnetosphere (SSC), there is an increase in the solar wind speed, a negative excursion of the Bz component of IMF, an increase in North and South polar cap indices, an increase in the AE index and a growth phase of Dst (compression phase) followed by a sharp decrease (main phase of the storm). We notice that for the case of March 2013, Dst presents a sudden impulse (SI) on March 15, 2013.

**Figure 4:**
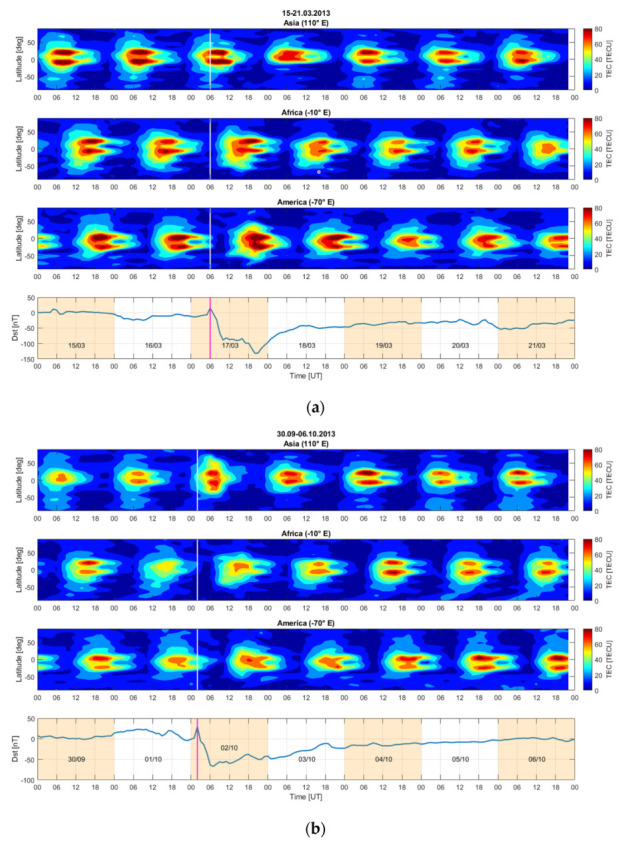
VTEC from GIM representing: Asian sector (top panel), African sector (second panel) and American sector (third panel). The Dst index is also indicated (bottom panel), (**a**) storm of 17 March 2013, (**b**) storm of 2 October 2013.

Figure 4a,b show VTEC maps in the three longitude sectors: Asian (110° E), African (−10° E) and American (−70° E). At the bottom of each figure, variations of Dst are plotted. The VTEC maps cover the period of two days before the storm and four days after the SSC, which is indicated with a white vertical line. For the storm of 17 March 2013 (Figure 3a), we observe a decrease in VTEC at the magnetic equator in the Asian sector, an increase in VTEC in the African sector over the northern crest of the equatorial ionization anomaly (EIA) and an increase in the VTEC on the two crests of the EIA in the American sector. On the second day of the storm, the VTEC decreases in all the longitude sectors.

The study of these three equinoctial storms that began between 02:00 and 6:00 UT highlights a similar pattern:

1. VTEC decreases in Asia and increases in Europe and America on the day of the storm.
2. VTEC decreases in all the longitude sectors the second day of the storm.

There is however an exception for the storm of 2 October 2013 for which VTEC increases the first day of the storm in Asia. This can be explained by the time difference in the onset of the storms: 02:00 UT on October 2013, 04:45 UT on 17 March 2015 and 06:00 UT on 17 March 2013. The times 02:00, 04:45 and 06:00 UT correspond to 09:00, 11:45 and 13:00 LT in Asia. If we consider that the disturbance carried by thermospheric winds takes roughly 3 to 4 h to arrive to middle and low latitudes [4,25], this gives times of arrival of the disturbance between 12.00–13.00 LT for the case of 2 October 2013, 14:45–15:45 LT for 17 March 2015 and 16:00–17:00 LT for 17 March 2013. On 2 October 2013 the thermospheric disturbance affects Asia at the time of the maximum of VTEC and maybe during the VTEC growth phase, while for storms of March 2015 and March 2013, the thermospheric disturbance impacts Asia when the VTEC is in phase of decay.

## 4. Results

**Figure 5:**
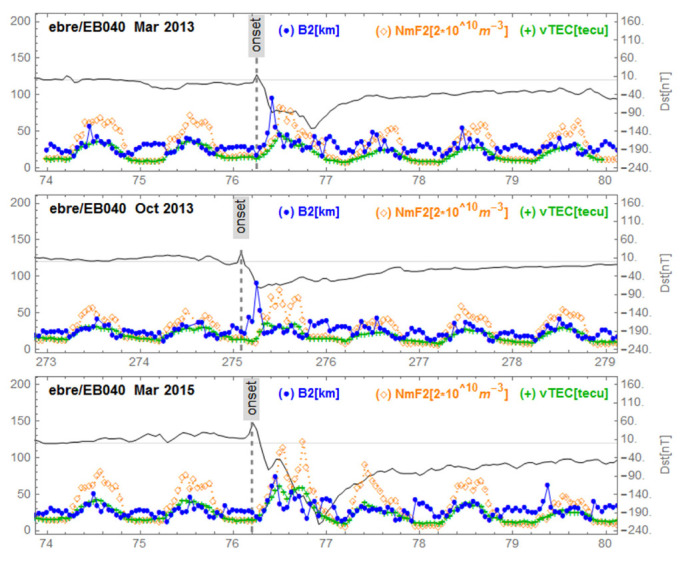
Variations of B2, NmF2 and VTEC at Ebre station during the three equinoctial storms 17 March 2013, 2 October 2013 and 17 March 2015. On each panel the Dst is superimposed.

**Figure 6:**
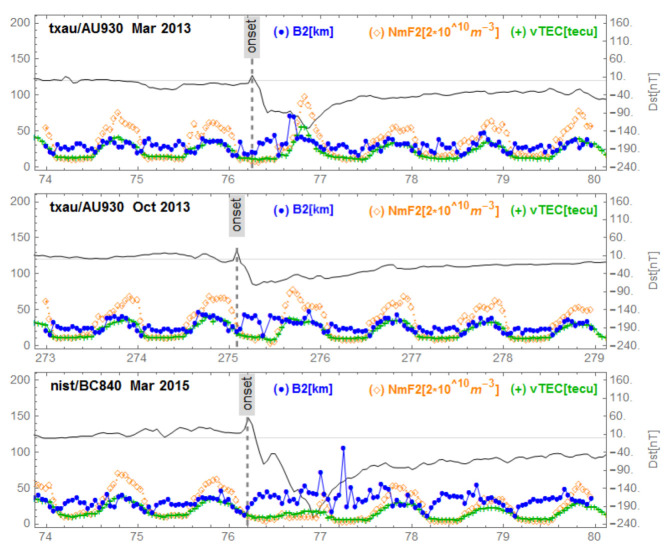
Variations of B2, NmF2 and VTEC for the stations Austin/Boulder during the three equinoctial storms 17 March 2013, 2 October 2013 and 17 March 2015. On each panel the Dst is superimposed.

**Figure 7:**
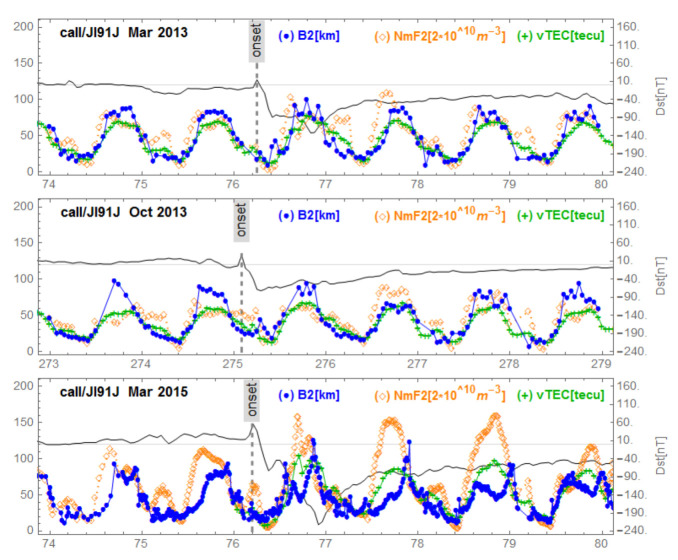
Variations of B2, NmF2 and VTEC for the station Jicamarca during the three equinoctial storms 17 March 2013, 2 October 2013 and 17 March 2015. On each panel the Dst is superimposed.

**Figure 8:**
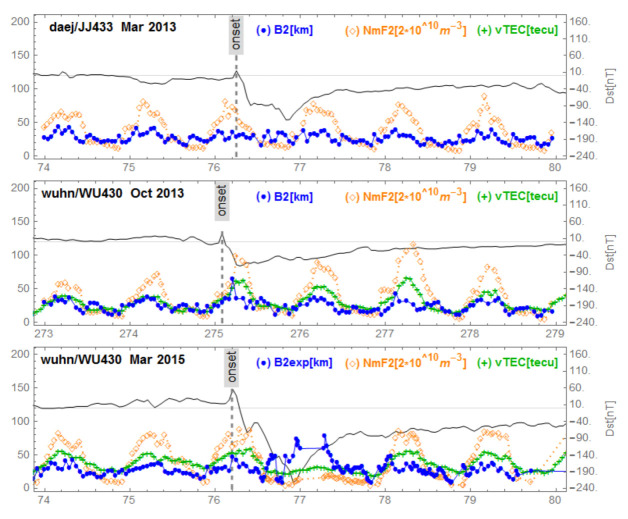
Variations of B2, NmF2 and VTEC for the stations I-cheon/Jeju/Wuhan during the three equinoctial storms 17 March 2013, 2 October 2013 and 17 March 2015. On each panel the Dst is superimposed.

**Figure 9:**
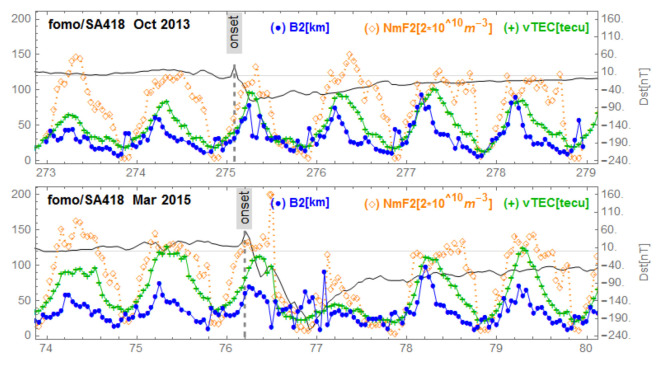
Variations of B2, NmF2 and VTEC at Fomo/ Sanya stations during the equinoctial storms of 2 October 2013 and 17 March 2015. No available data for 15 March 2013.

Figure 5, Figure 6, Figure 7, Figure 8 and Figure 9 show variations of NmF2, VTEC and B2 for the three storms studied, for representative stations in different regions. Figure 5 corresponds to mid-latitudes of the European-African longitude sector. Figure 6 and Figure 7 correspond to middle and low latitudes, respectively, of the American longitude sector. Figure 8 and Figure 9 correspond to mid-latitudes and low latitudes, respectively, of the Asian longitude sector. In Figure 9 data concerning the storm of 17 March 2013 are missing. On the panels of each figure the Dst magnetic index is superimposed.

Figure 5, corresponding to Ebre observatory, shows the same signature for the three geomagnetic storms. We observe a post SSC peak of B2 preceding storm-related VTEC and NmF2 peaks. We identified this behavior as ”B2 spike”. In the Figure 6, corresponding to the mid-latitudes in the American sector, we observe the same behavior for the storm of 17 March 2013 (top panel) which had positive effects. The middle panel of Figure 6 reports the series under de 2 October 2013 with no particular effects. On the bottom panel of Figure 6 (at Boulder), corresponding to mid-latitudes in the American sector for the great storm of 17 March 2015, we observe a fluctuating behavior of B2 associated with decreases in NmF2 and VTEC; we call this a “complex case”. In Figure 7 corresponding to the observatory of Jicamarca, located at the geomagnetic equator in the American sector, we observe, as in the case of Ebre (Figure 5), the same signature for the storms: the peak of B2 followed by enhancements of VTEC and NmF2 partially masked by the diurnal behavior of both VTEC and NmF2 related to the development of the EIA. In Figure 8, corresponding to mid-latitudes in the Asian longitude sector, the signature of the B2 spike case can be seen for the storm of 2 October 2013 (middle panel), while for the storm of 17 March 2015 (bottom panel) the signature of a complex case can be seen. In the top panel corresponding to the storm of 17 March 2013, there is no remarkable effect of the storm. Finally, in Figure 9, corresponding to the low latitudes in the Asian longitude sector, the signature of a complex case for the great storm of 17 March 2015 (bottom panel) is noted. In the top panel of Figure 9 that corresponds to the storm of 2 October 2013, we observe a growth of B2 at the same time as NmF2 and VTEC on the storm days followed by a decrease in B2. The days after the minimum Dst we observe an increase in B2 associated with an increase in NmF2 and VTEC, indicating a particular case.

The results are summarized in Table 2. There are 14 cases with available data: eight out of 14 cases are B2 spike cases. These cases correspond to positive ionospheric storms; three cases out of 14 are complex cases (complex behavior of B2, decrease in NmF2 and VTEC), and they correspond to negative ionospheric storms.

All complex cases correspond to the great storm of 17 March 2015. There are other different cases with a decrease in B2 associated with a positive ionospheric storm during the weakest storm of 2 October 2013. There is also one location in the Asian mid-latitude sector without any effect on B2 and NmF2 during the storm of 17 March 2013.

### 4.1. St. Patrick Storm 2015 Results

It is noted that for the great storm of 17 March 2015 there are either cases with B2 spikes or complex cases. We have therefore extended the samples of data for this storm using manually scaled ionograms every 15 min, and the corresponding variations have been analyzed by sectors including additional stations.

The results summarized in Table 3 report the behavior observed in middle and low latitudes. As for the cases studied previously, we mostly observe two clear signatures: B2 spikes and/or complex cases. In the case of the station of Grahamstown, we observe a spike case followed by a complex case. The complete set of plots for this storm by sector are not shown in the paper and are provided in the Supplementary Materials. In those figures the B2 and VTEC peaks considered in the time analysis are marked.

### 4.2. B2 Time Response Analysis

**Figure 10:**
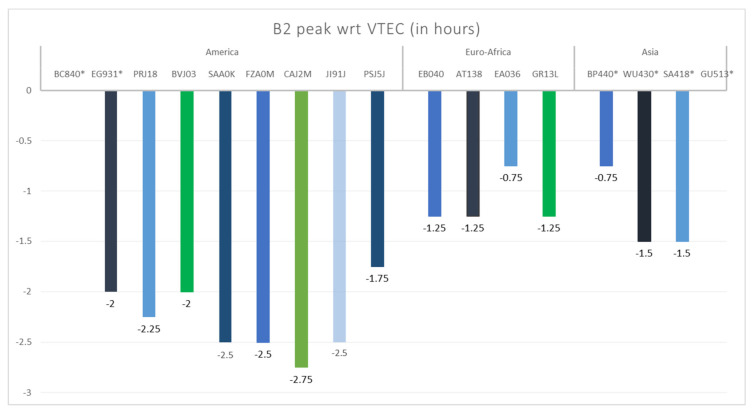
Time delay analysis results between experimental B2 and GPS VTEC during the 24 h (UT) of the St. Patrick 2015 storm main phase by regions. Indicated is the difference in time of the B2 spike occurrence with respect to the peak in VTEC. Complex and particular cases as well as those with gaps are marked with (*).

The data series that present B2 spikes during the St. Patrick 2015 storm were subsequently analyzed and cross-correlated to investigate the time of response of the different measurements according to location. For each series of observations, the peaks of the analyzed parameters (B2, VTEC and NmF2) were identified starting from the SSC occurrence. Figure 10 shows the results of the cross-correlation analysis between experimental B2 and VTEC during 24 h (UT) of the main phase of the St. Patrick 2015 storm, grouped by regions. The color bars indicate time differences (in hours) between B2 peaks and the corresponding maxima in VTEC. The stations with (*) represent complex cases or gaps in data (Boulder, Eglin and Asian stations). It is noted that the B2 parameter consistently presents a peak after the SSC and before VTEC in a range from 45 min to almost 3 h depending on the sector and latitude. The shortest response times are found at Asian and European mid-latitude stations (Beijing and San Fernando) with a delay of 45 min between B2 and VTEC peaks. The longest time of VTEC reaction to the SSC took place at South American stations Jicamarca, San Luis and Fortaleza with 2.5 h and Cachoeira Paulista with 2.75 h.

## 5. Discussion

Taking into account all the analyzed cases, we have a total of 26 observation series: 16 with B2 spike cases (62%), 6 complex cases (23%) and 4 considered undefined due to data gaps and/or lack of effects (15%).

The analysis of B2, NmF2 and TEC parameter series during equinoctial storms showed two main types of behavior. The B2 spike case, meaning a peak on B2 just prior to NmF2 and VTEC storm-related enhancements, have been found to correspond to positive ionospheric storm effects. The complex case with a chaotic behavior of B2 associated with a decay of NmF2 and VTEC corresponds to negative ionospheric storm effects. The behavior found in most cases corresponds to the action of different dominant and, at the same time, competing mechanisms. During a positive ionospheric storm there are modifications due to transport of ionization by winds and electric fields [26]. Equatorward blowing neutral winds from high latitudes tend to push the plasma up along the magnetic field lines, at heights where the recombination is slow, and as a consequence the plasma density increases [5,27]. The depression in ionization that characterizes the negative storms is thought to be connected with changes in neutral composition at ionospheric heights. This in turn decreases the O/N2 ratio, therefore increasing the ionospheric loss coefficient [27,28]. It follows then that stations located within these areas of low O/N2 ratio will exhibit negative phases during storms, as observed by [7]. This is in line with our results for the Asian mid- and low latitude stations (Beijing, Wuhan, Sanya) and in the American mid-latitude stations (Boulder, Eglin) for the storm of St. Patrick 2015. As reported by [24], data from Global Ultraviolet Imager (GUVI) revealed a large decrease in O/N2 ratio along the Asian region and over some mid-latitudes of the American sector during the days 17–18 March 2015.

## 6. Conclusions

The variations of the NeQuick bottomside thickness parameter B2, computed with experimentally derived NmF2 and (dN/dh)max, were analyzed during three ionospheric storms caused by CMEs which occurred during the same season (equinox). The analysis of the B2 along with vertical TEC and maximum electron density (NmF2) variations over more than 20 stations at middle and low latitudes of different longitude sectors (Asia, Euro-Africa and America) before, during and after the geomagnetic events shows two kinds of responses: (1) a peak of B2 parameter after SSC and prior to the storm-related VTEC and NmF2 peaks (in ~60% of the cases) and (2) a fluctuating B2 associated with decrease in VTEC and NmF2 (~25% of the cases). Few observations (~15%) correspond to stations where the ionosphere does not appear to be affected by the storms or those which represent data gaps.

The behavior observed has been related to the dominant factor acting after the CME shocks, i.e., storm-driven neutral winds push ion and neutral species up or down along the magnetic field lines, causing positive and negative storm effects.

The analysis of the response time in different measurements according to location for the St. Patrick 2015 storm shows that B2 reacts before VTEC after an SSC in all the analyzed cases in a time range from 45 min to almost 3 h.

The assimilation of storm time experimentally derived thickness parameter B2 into empirical models to improve the ability of such models to be adapted to different geomagnetic conditions is planned to be investigated. Such investigation is in line with the results obtained by [14,15].

These results demonstrate the importance of simultaneous observations of electron density profiles by ionosondes and GNSS derived VTEC to understand the physical processes that control ionosphere variability particularly under geomagnetic storm conditions.

## Figures and Tables

**Table 1:** Data used in the study.

Parameters	Source/Description
Sun observations	

Smoothed sunspot number	WDC-SILSO Royal Observatory of Belgium, Brussels
Smoothed 10.7 cm solar radio flux	http://www.sidc.oma.be./silso/home (accessed on 15 September 2021)
	Solar and Heliospheric Observatory (SOHO)
Detection of CME	http://www.nasa.gov/mission_pages/soho (accessed on 15 September 2021)
Solar wind parameters	Omniweb
	http://omniweb.gsfc.nasa.gov/ (accessed on 15 September 2021)
Vx	Eastward component of the Solar Wind
$E_{m}={\;V}_{x}\cdot B_{T}\cdot \sin^{2}\left(\theta /2\right)$	Merging electric field
$B_{T}=\sqrt{B_{y}{}^{2}+B_{z}{}^{2}}$	
V_x_	Solar wind velocity
B_T_, B_y_, B_z_	Total, y and z- GSM components of the IMF
Θ	polar angle of transverse IMF vector
Magnetospheric parameters	World Data Center for Geomagnetism, Kyoto
	http://wdc.kugi.kyoto-u.ac.jp (accessed on 20 September 2021)
Dst magnetic index	Dst gives information on the different phases of the storm
Auroral magnetic index, AE	AE is used to evaluate energy deposited in the auroral zone
Polar Cap magnetic index North and South, PCN, PCS	International Service of Geomagnetic Indices
	http://isgi.unistra.fr/ (accessed on 20 September 2021)
Ap	PC is a proxy of the merging electric field (Em)
Am	Worldwide Magnetic activity
	http://isgi.unistra.fr/ (accessed on 20 September 2021)
Ground datasets	Center for Orbit Determination in Europe (CODE)
	http://ftp.aiub.unibe.ch/CODE/ (accessed on 13 April 2021)
Global ionospheric maps, GIMs	
	Digital ionogram database (DIDBase)
Ionograms	http://umlcar.uml.edu/DIDBase/ (accessed on 23 October 2021)
	Manually scaled ionograms
VTEC from GNSS	
	ftp://data-out.unavco.org (accessed on 10 June 2021)
	ftp://igs.bkg.bund.de/EUREF/ (accessed on 10 June 2021)
	ftp://ftp.sonel.org/gps/data (accessed on 10 June 2021)
	ftp://www.ngs.noaa.gov/cors/rinex (accessed on 10 June 2021)

**Table 2:** Variations of B2, NmF2 and VTEC observed at middle and low latitudes stations in the different longitude sectors and for the three storms 17 March 2013, 2 October 2013 and 17 March 2015.

Storm/ Region	17 March 2013 Intense Storm Min Dst => −132 nT	2 October 2013 Moderate Storm Min Dst => −72 nT	17 March 2015 Great Storm Min Dst => −222 nT
European-African Mid-latitudes	Positive storm + NmF2 + B2 spike	Positive storm + NmF2 + B2 spike	Positive storm + NmF2 + B2 spike
American Mid-latitudes	Positive storm + NmF2 +B2 spike	No effect	Negative storm Complex case
American Low latitudes	Positive storm + NmF2 + B2 spike	+B2 spike + NmF2	Positive storm + NmF2 + B2 spike
Asian Mid-latitudes	No changes in NmF2 No effect	Positive storm + NmF2 + B2 spike	Negative storm Complex case
Asian Low latitudes	No data	Decrease in NmF2 Particular case	Negative storm Complex case

**Table 3:** Variations of B2, NmF2 and VTEC observed in middle latitude stations only for the storm of 17 March 2015. (**a**) Mid-latitude stations and (**b**) Low latitude stations.

Location/Station Code	17 March 2015 Min Dst => −222 nT
(**a**)	
Asian Mid-latitudes Beijing BP440	Negative storm Complex case
Asian Mid-latitudes Wuhan WU430	Negative storm Complex case
European-African Mid-latitudes Arenosillo EA036	Positive storm + NmF2 + B2 spike
Europeand-African Mid-latitudes Athens AT138	Positive storm + NmF2 + B2 spike
European-African Mid-latitudes Grahamstown GR13L	Positive storm + NmF2 + B2 spike Followed by Negative Storm − NmF2 Complex case
American Mid-latitudes Boulder BC840	Negative storm − NmF2 Complex case
American Mid-latitudes Eglin EG931	Negative storm − NmF2 Complex case
American Mid-latitudes Ramey PRJ18	Positive storm + NmF2 + B2 spike
American Mid-latitudes Port Stanley PSJ5J	Positive storm + NmF2 + B2 spike
(**b**)	
America Low latitudes Boa Vista BVJ03	Positive storm + NmF2 +B2 spike
America Low latitudes Sao Luis SAA0K	Positive storm + NmF2 +B2 spike
America Low latitudes Fortaleza FZA0M	Positive storm + NmF2 + B2 spike
Asia Low latitudes Guam GU513	Negative storm Complex case data gaps
Asia Low latitudes Sanya SA418	Negative storm Complex case

## Data Availability

The data supporting reported results, including links to publicly archived datasets, can be found in Table 1.

## Supplementary Materials

The following are available online at https://www.mdpi.com/article/10.3390/s21217369/s1, Figure S1: B2, NmF2 and VTEC variations at American stations during the 17 March 2015 storm. B2 and VTEC peaks considered in the time analysis are marked in magenta and cyan, respectively. Figure S2: B2, NmF2 and VTEC variations at Asian stations during the 17 March 2015 storm. B2 and VTEC peaks considered in the time analysis are marked in magenta and cyan, respectively. Figure S3: B2, NmF2 and VTEC variations at European-African stations during the 17 March 2015 storm. B2 and VTEC peaks considered in the time analysis are marked in magenta and cyan, respectively.
