# Supplementary material for: B2 Thickness Parameter Response to Equinoctial Geomagnetic Storms
Source: Sensors (Basel). 2021 Nov 5;21(21):7369. doi: 10.3390/s21217369 (PMC8588179; doi:10.3390/s21217369)
Supplement: Supplementary file 1 [file sensors-21-07369-s001.zip › sensors-1421616-supplementary.pdf]

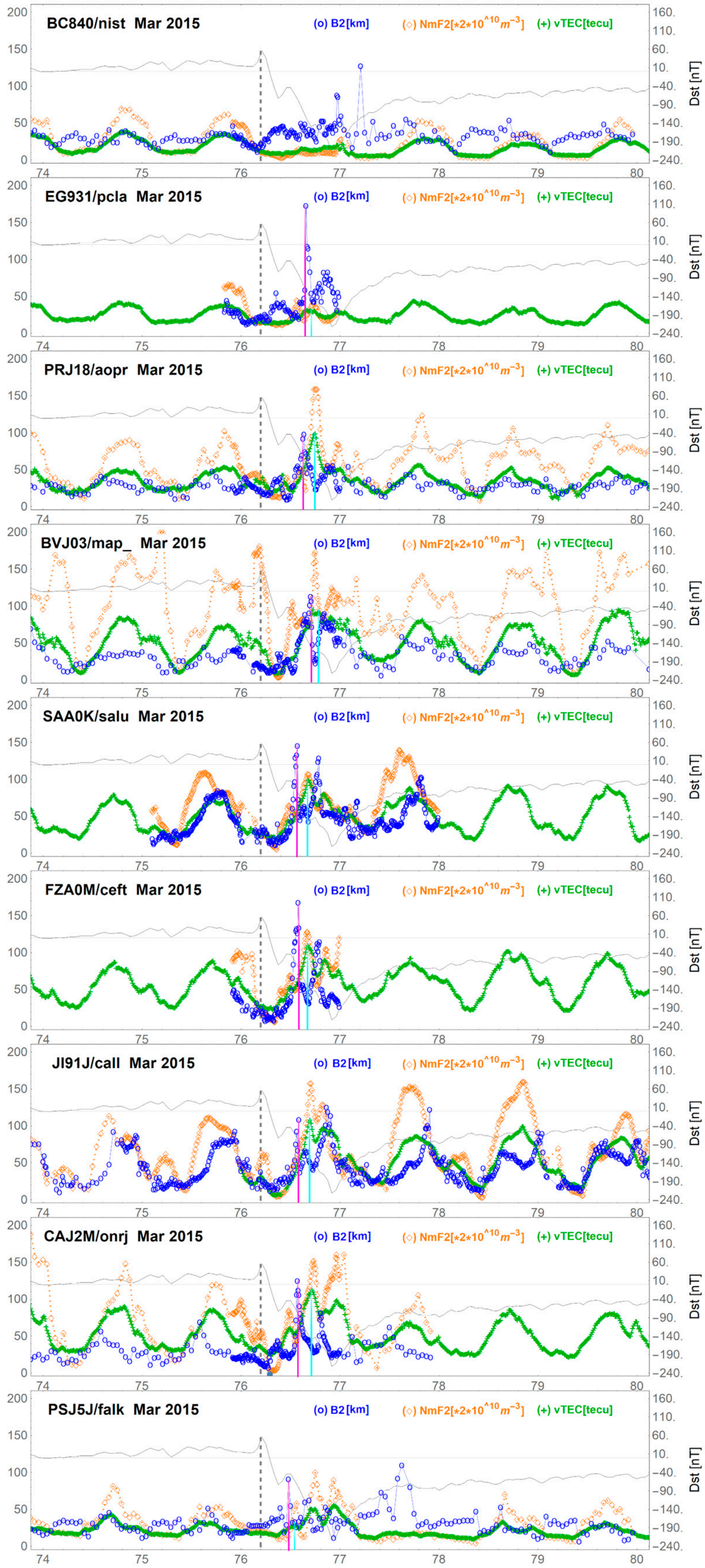

Figure S1: B2, NmF2 and VTEC variations at American stations during the 17 March 2015 storm. B2 and VTEC peaks considered in the time analysis are marked in magenta and cyan, respectively.

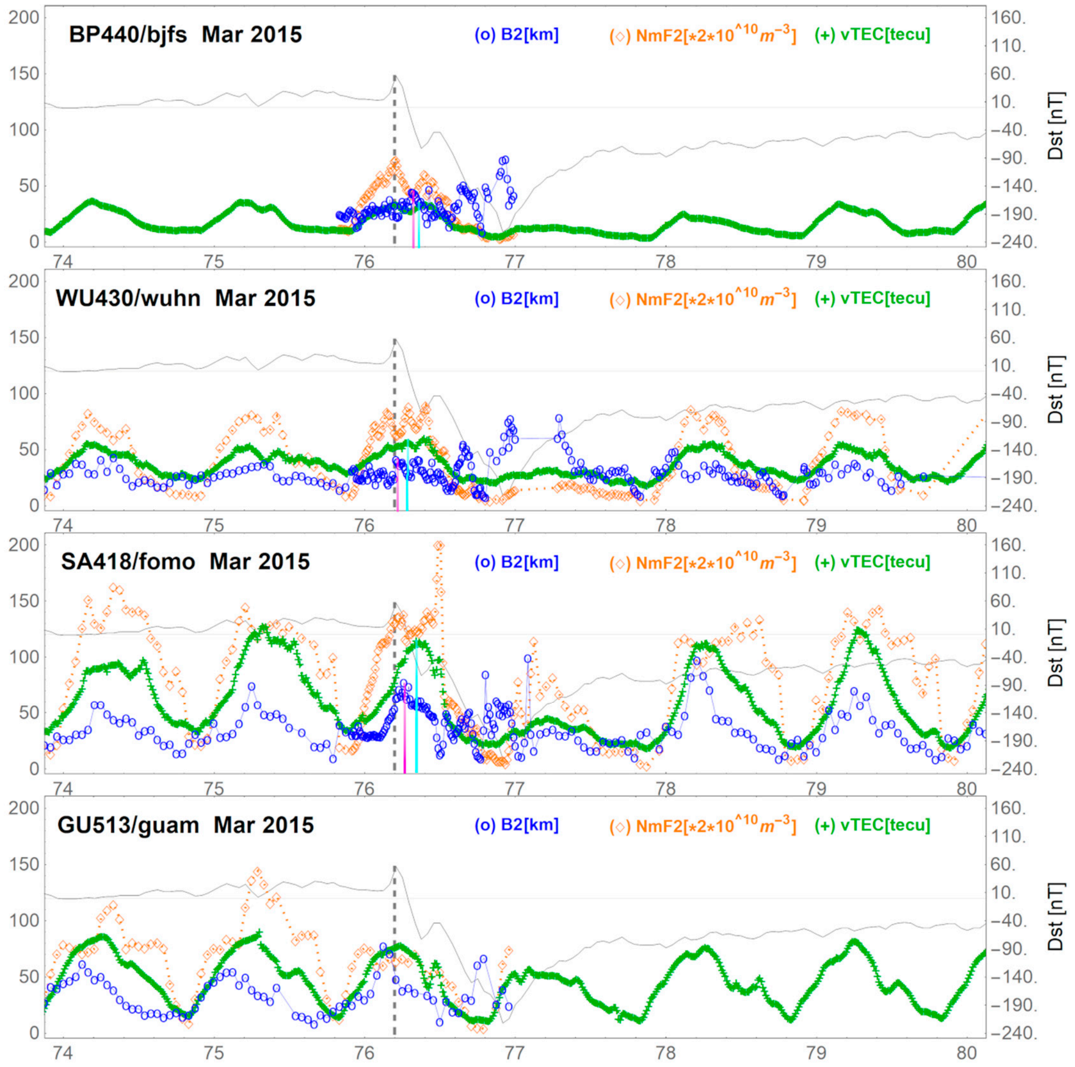

Figure S2: B2, NmF2 and VTEC variations at Asian stations during the 17 March 2015 storm. B2 and VTEC peaks considered in the time analysis are marked in magenta and cyan, respectively.

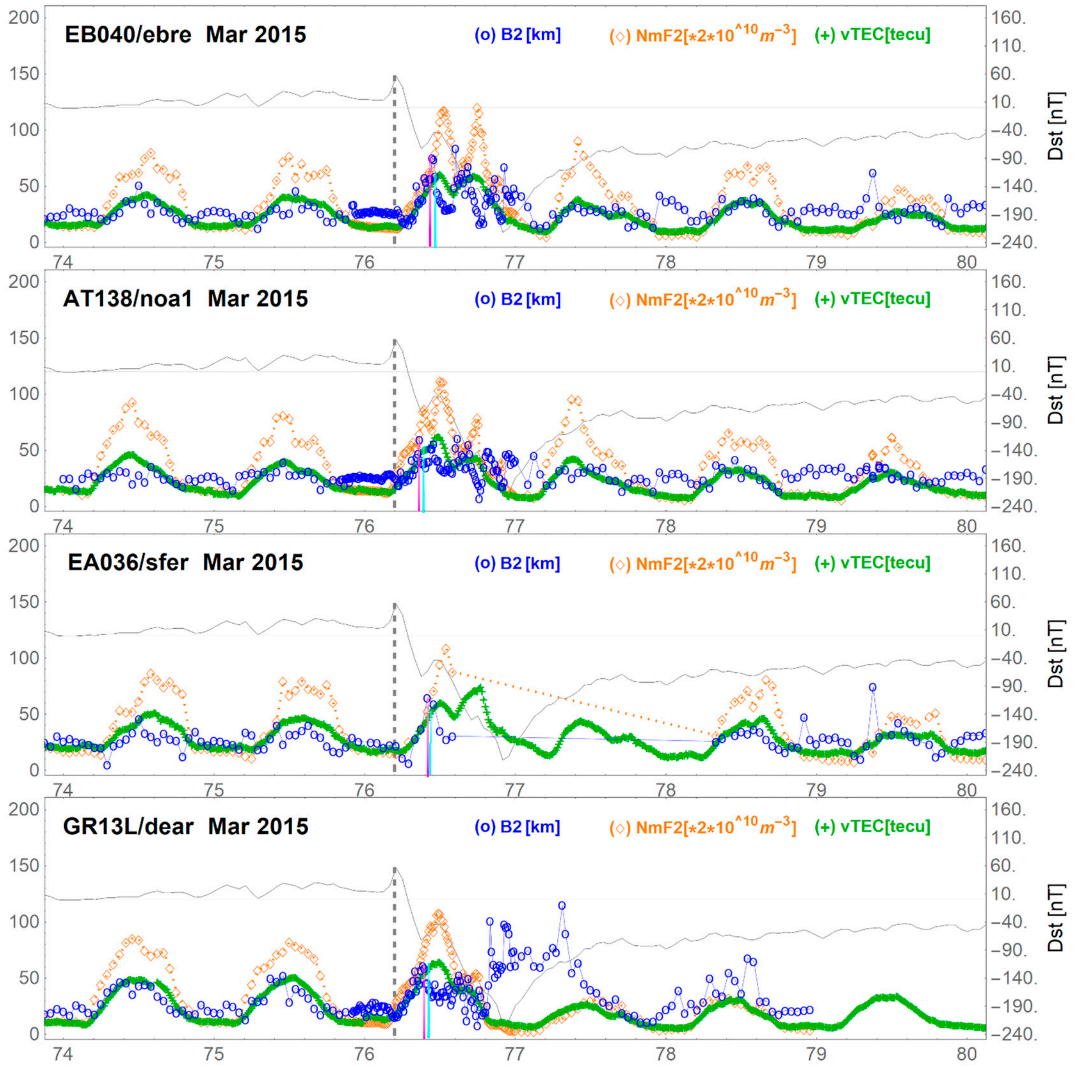

Figure S3: B2, NmF2 and VTEC variations at European-African stations during the 17 March 2015 storm. B2 and VTEC peaks considered in the time analysis are marked in magenta and cyan, respectively.
